# Anti-methicillin-resistance *Staphylococcus aureus* (MRSA) compounds from *Bauhinia kockiana* Korth. And their mechanism of antibacterial activity

**DOI:** 10.1186/s12906-018-2137-5

**Published:** 2018-02-20

**Authors:** Yik Ling Chew, Adlina Maisarah Mahadi, Kak Ming Wong, Joo Kheng Goh

**Affiliations:** 1 Faculty of Pharmaceutical Sciences, UCSI University, No. 1 Jalan Menara Gading, UCSI Heights, 56000 Kuala Lumpur, Malaysia; 2 School of Science, Monash University Malaysia, Jalan Lagoon Selatan, 47500 Bandar Sunway, Selangor Darul Ehsan Malaysia

**Keywords:** *Bauhinia kockiana* Korth, Alkyl gallates, MRSA, Scanning electron microscopy

## Abstract

**Background:**

*Bauhinia kockiana* originates from Peninsular Malaysia and it is grown as a garden ornamental plant. Our previous study reported that this plant exhibited fairly strong antioxidant and antimicrobial activities. This paper focused on the assessment of the antibacterial activity of *B. kockiana* towards methicillin-resistance *Staphylococcus aureus* (MRSA), to purify and to identify the antibacterial compounds, and to determine the mechanism of antibacterial activity.

**Methods:**

Antibacterial activity of *B. kockiana* flower was evaluated qualitatively and quantitatively using disc diffusion assay and microbroth dilution method. Minimum inhibitory concentration (MIC) and minimum bactericidal concentration (MBC) of extracts were examined. Phytochemical analysis was performed to determine the classes of phytochemicals in the extracts. Bioactivity guided isolation was employed to purify the antibacterial agents and identified via various spectroscopy methods. Scanning electron microscopy (SEM) technique was used to evaluate the antibacterial mechanism of extract and compounds isolated.

**Results:**

*B. kockiana* flower was found to exhibit fairly strong antibacterial activity towards both strains of MRSA bacteria used, MIC varies from 62.5–250 μg/mL. Tannins and flavonoids have been detected in the phytochemical analysis. Gallic acid and its ester derivatives purified from ethyl acetate extract could inhibit MRSA at 250–500 μg/mL. SEM revealed that the cells have undergone plasmolysis upon treatment with the extract and compounds.

**Conclusion:**

Tannins and polyphenols are the antibacterial components towards MRSA in *B. kockiana*. Massive leakage of the cell content observed in treated cells showed that the phytochemicals have changed the properties of the cell membranes. Amphiphilic nature of the compounds exhibited the antibacterial activity towards MRSA via three stages: (1) cell membrane attachment; (2) cell membrane fluidity modification; and (3) cell membrane structure disruption.

**Electronic supplementary material:**

The online version of this article (10.1186/s12906-018-2137-5) contains supplementary material, which is available to authorized users.

## Background

Antibiotic resistance is known as the inability of antibiotic to produce effects on the bacteria. Bacteria develop resistance towards antibiotic through overuse or misuse of antibiotics. Antibiotic-resistant bacterial infections have been widely spread around the world. Number of cases for antibiotic resistance is increasing, as seen in massive increment in morbidity and mortality rate caused by infectious diseases. Public health organizations such as Word Health Organization and Centre of Disease Control have declared that the crisis of antibiotic resistance is becoming worse as we are living in the “post –antibiotic era” [1]. One of the very serious and commonly occurred antibiotic resistant bacteria is methicillin-resistance *Staphylococcus aureus* (MRSA). According to Gross and Golkar, Bagazra and Pace, MRSA causes death more than human immunodeficiency virus (HIV) or acquired immune deficiency syndrome (AIDS), emphysema, homicide and Parkinson’s disease [2, 3]. *S. aureus* is an asymptomatic carrier found on the skin surface of human. It can develop into an opportunistic pathogen and causing infections to any wound and opening of the skin. MRSA has evolved and developed resistance towards the β-lactam antibiotics which are previously used as antibacterial agents, such as methicillin, penicillin, oxacillin, amoxicillin and cephalosporins [4]. MRSA develops resistance towards antibiotics by adapting themselves to the mode of action of β-lactam antibiotics [5]. Now, the first line antibiotic to treat MRSA infection is vancomycin. The dosage of vancomycin used in treatment has to be properly monitored, where the dosage should not exceed 2 g in any 24-h period. Overdose of vancomycin may cause “red man syndrome”, a hypersensitivity reaction linked to rapid administration of the antibiotic [6]. In addition, the emergence of first clinical infection with MRSA which is resistant to vancomycin – vancomycin resistant *S. aureus* (VRSA) on July 2002 [7]. and the multidrug resistance bacteria in European hospitals have globally alarms us that new candidate of antibiotic is needed since the treatment options for infected patients are extremely limited at the moment [8].

Plants synthesized secondary metabolites as a chemical defence system against predation by herbivores, insects and microorganism [9]. The bioactive secondary metabolites from different classes of phytochemicals could kill or inhibit the microorganism growth via different mechanisms. For instance, epicatechin gallate inhibits MRSA by insertion into the bacteria cytoplasmic membrane and disruption of penicillin-binding protein 2a–mediated β-lactam resistance [10]; berberine and piperine could interfere the microbial growth by intercalating the cell wall and DNA [11, 12]; and tannins inhibit the growth of *Klebsiella pneumoniae* by damaging the bacterial cell membranes and causing cell swelling.

**Fig. 1:**
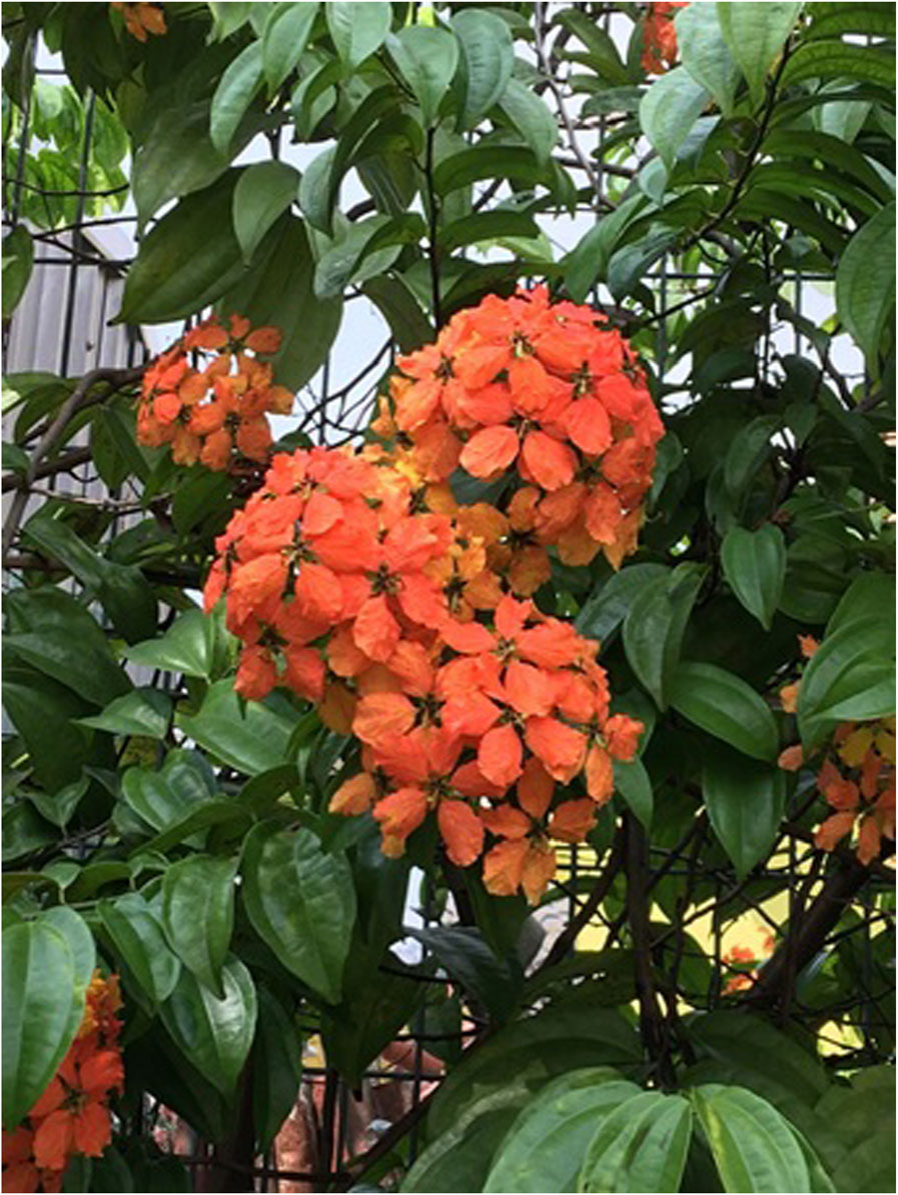
*Bauhinia kockiana* Korth with its bright orange-red magnificent inflorescences

Plants from Leguminosae family are well known for their medicinal properties. Leguminosae is divided into three subfamilies which are Mimosoideae, Caesalpinioideae and Faboideae. *Bauhinia* species are from Caesalpinioideae subfamily of Leguminosae. *Bauhinia kockiana* is a plant originates from Peninsular Malaysia but now it can also be found in the tropical forests of Thailand and Sumatra [13]. It is usually grown in the garden as ornamental plant because of its bright orange-red colour (Fig. 1) [14, 15]. This plant is used as ethnomedicine for treating various diseases. In Sarawak, Eastern Malaysia, the roots of *B. kockiana* is used by *Kelabit* ethnic group for treating gonorrhoea, nervous debility, insomnia as well as fatigue [16, 17]. The bark and root infusions are used to treat toothache [14]. Researches on the bioactivities and phytochemicals of *B. kockiana* are relatively new and not.

many literatures are available. Our previous studies have reported that *B. kockiana* could exhibit anticancer activity towards various types of cell lines, including MCF-7, PC-3 cells, LNCaP, HCT-116 and DU145 cells [13]. Antioxidant activities of *B. kockiana* has also been studied. Our studies reported that *B. kockiana* flowers and leaves consisted very high total phenolic content, 4440 to 7540 mg gallic acid equivalent/100 g, and exhibited strong free radical scavenging and ferric reducing antioxidant power [14, 15].

The main objectives of this reports are to evaluate the antibacterial activity of *B. kockiana* flowers towards MRSA, to purify and identify the antibacterial compounds from *B. kockiana* flowers and to evaluate the antibacterial mechanism of the compounds using scanning electron microscopy (SEM). This is the first report on the antibacterial compounds against MRSA found in *B. kockiana* flowers, and the mode of actions of the extracts and compounds are also investigated and evaluated using SEM.

## Methods

### Plant materials and preparation of extracts

The fresh flowers of *B. kockiana* were collected from a private garden in Saujana Impian, Kajang, Selangor, Malaysia. The owner of the garden, Ms. Lu Yii Ying presented the flower samples to authors as a gift. She has interest in understanding in the scientific information of the plant which will be shared with her. The plant materials were collected on the day when extraction was performed. Voucher specimen (MUM-LEGUM-001) was deposited in the herbarium of School of Science, Monash University Malaysia. The extraction of flowers was performed as according to Chew et al. [13] with slight modifications. Fresh flowers of *B. kockiana* (1.7 kg), were collected and washed with distilled water, then freeze-dried using a freeze dryer. The dried flowers (550 g), were powdered and percolated sequentially with 12 L of solvents in the following order: hexane, dichloromethane, ethyl acetate and methanol. The plant material was soaked for 48 h in each solvent and the extraction was repeated for several times with fresh solvent until successive extraction was achieved. The hexane, dichloromethane, ethyl acetate and methanol extracts were dried individually using a rotary evaporator at 40 °C. The extracts were kept at − 20 °C until further analysis.

### Fractionation and isolation of bioactive compounds

**Fig. 2:**
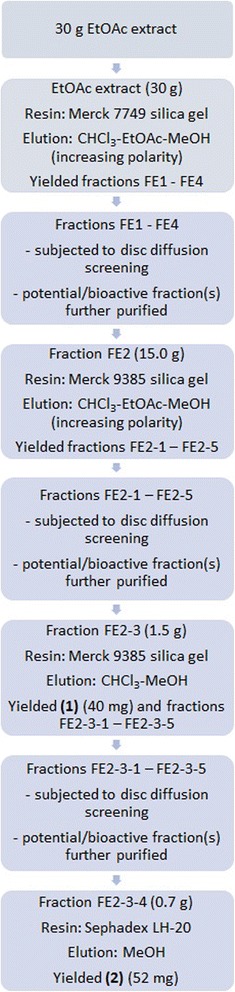
Schematic diagram of fractionation and isolation of compound **1** and **2**

Bioactivity guided isolation was employed in this study to purify the antibacterial agents. Fractions yielded from various chromatography were evaluated using disc diffusion assay. Fraction(s) which exhibited potential antibacterial activity were further purified to yield a single compound. 30 g of ethyl acetate extract was fractionated with vacuum liquid chromatography over Merck 7749 silica gel with chloroform - ethyl acetate - methanol (in increasing polarity), as eluents. The polarity of solvents was increased to yield four major fractions, labelled FE1 - FE4. All fractions were subjected to disc diffusion assay. A part of fraction FE2 (15.0 g) was separated using Merck 9385 silica gel and eluted in a gradient manner with chloroform-ethyl acetate-methanol, in increasing polarity to yield five fractions (FE2–1 - FE2–5), and these fractions were subjected to disc diffusion assay. Fraction FE2–3 (1.5 g) was further purified in a similar manner using a silica gel column using Merck 9385 silica gel with stepwise gradient elution with chloroform-methanol and yielded **1** (40 mg), and 5 other fractions (FE2–3-1 – FE2–3-5). Fraction FE2–3-4 (0.7 g) was further purified using Sepahadex LH-20 and eluted with methanol to give rise to **2** (52 mg) (Fig. 2). The purities of **1** and **2** were confirmed using reversed-phase high performance liquid chromatography; the molecular masses were obtained using gas chromatography coupled with mass spectrometry and their chemical structures are characterized using nuclear magnetic resonance (NMR) and fourier transform infra-red (FTIR) spectroscopy methods.

### Phytochemical analysis

Phytochemical screening for flavonoids, tannins and steroids were performed as described previously [15]. Flavonoids were detected using magnesium turnings; tannins were detected using ferric chloride (0.01 g/mL) method; and presence of steroids was confirmed using chloroform-concentrated sulphuric acid mixture.

### Antibacterial activity

#### Bacteria strains

Bacteria used in this study were MRSA ATCC 33591 and MRSA clinical isolate. MRSA clinical isolate obtained from Universiti Putra Malaysia.

#### Preparation of innoculums

One single colony of each type of microorganism (from the nutrient agar stock culture) was inoculated with a sterile loop, and is transferred into 10 mL sterile nutrient broth (Oxoid). The broth cultures were incubated in a shaking incubator at 37 °C for 16–20 h.

#### Antibacterial susceptibility test: Disc diffusion assay

**Fig. 3:**
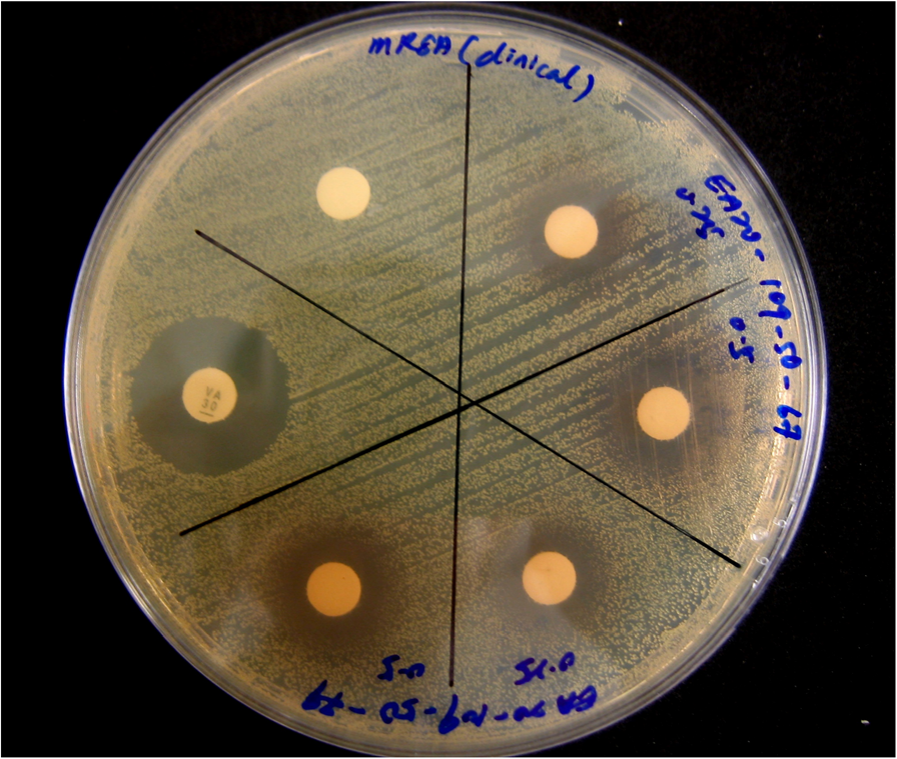
Inhibition zone of fractions exhibit antibacterial activity in disc diffusion assay

The antibacterial assay was performed using disc diffusion (Kirby-Bauer) method as described by Chew et al. [15]. This is a qualitative assay which is commonly performed to evaluate the antimicrobial activity of phytochemicals or extracts. Briefly, the density of bacteria was standardized to 1 × 10^8^ coliform units (cfu)/mL using Miles and Misra technique [18] and was swabbed onto Mueller Hinton Agar (Oxoid) surface. 1 mg of crude extract or 0.5 mg of fractions were dissolved initially in 100 μL methanol and loaded onto sterile blank disc (6 mm diameter; Oxoid). The discs were then impregnated onto inoculated agar. 30 μg vancomycin (Oxoid) and blank disc loaded with 100 μL methanol without extract or fraction were served as positive and negative controls, respectively. The plates were left at 4 °C for an hour to allow the diffusion of extracts before they were incubated for 16–20 h at 37 °C. Antibacterial activity was indicated when clear inhibition zones observed around the discs. The diameter of the inhibition zones was measured (Fig. 3) and the results were expressed as mean of three independent experiments. The test was repeated three times.

### Microbroth dilution method: Determination of minimum inhibitory concentration (MIC) and minimum bactericidal concentration (MBC)

The crude extracts and compounds which showed positive inhibition activity in the disc diffusion method were subjected for quantitative evaluation: MIC and MBC determination. The MIC assay was done using microbroth dilution method, as described by the Clinical and Laboratory Standards Institute [19]. Each treatment with extracts or compounds and the controls were performed in triplicates. 1 mg of extracts and compounds were dissolved in 10 μL dimethyl sulfoxide (DMSO) in H_2_O to yield 1000 mg/mL extracts or compounds, which were further diluted to 1 mg/mL. A serial two fold dilution was mixed with nutrient broth in 96-well microtitre plates to give a final concentration of 0.02–1.00 mg/mL. 100 μl of 24-h bacteria suspension of 1 × 10^6^ cfu/mL was applied to nutrient broth supplemented with extracts or compounds. Vancomycin (Sigma Aldrich) was used as the positive control, while 0.1% DMSO was added into the negative control wells. The microtitre plate was incubated at 37 °C for 24 h. MIC was recorded as the lowest concentration of extracts or tested compounds which completely inhibited bacteria growth. MBC was determined when no visible growth seen on the first streak on MHA of the clear wells. The test was repeated three times.

### Scanning electron microscopy (SEM) analysis

SEM analysis was performed according to Chan et al. [20] with slight modifications. Bacterial suspension was tested with 2 × MIC of extracts and compounds in sterile microcentrifuge tubes, incubated at 37 °C for 6 h. The treated bacteria were then centrifuged at 5000 rpm for 3 mins, and washed thrice with phosphate buffer saline (PBS). 20 μL of the bacterial suspension was pipetted onto a glass slide and was left to air-dry. The slides (control and treated) were fixed with 2.5% glutaraldehyde for 24 h, and were later washed with PBS. The slides were then immersed in increasing concentration of ethanol (10, 20, 4, 80, 99.5 and 100%), each for 15 mins. The slides were dried overnight in the desiccator. The dried specimens were coated with a thin layer of platinum and were examined under a scanning electron microscope.

### Statistical analysis

All assays were carried out in triplicates. The experimental results for disc diffusion were expressed as mean ± standard deviation. The data were analyzed using one way analysis of variance (ANOVA) using SPSS version 20.

## Results

### Antibacterial activity of extracts toward MRSA and the phytochemical content

The qualitative analysis using disc diffusion assay showed that no inhibition in both hexane and dichloromethane extracts (Table 1). Ethyl acetate and methanol extracts exhibited antibacterial activity in disc diffusion assay, but no significant difference was noticed in the diameter of inhibition for both extracts on MRSA strains. Ethyl acetate extract displayed slightly stronger antibacterial activity than methanol, with lower MICs. Phytochemical analysis showed that tannins and flavonoids were present in the extracts (Table 2). This is also in agreement with our previous study on the crude extract [15], except that steroid was absent in both ethyl acetate and methanol extracts. Ethyl acetate showed lower MIC and MBC (MIC 62.5–125 μg/mL; MBC 125–500 μg/mL) compared to methanol (MIC 125–250 μg/mL; MBC 250–500 μg/mL). Both extracts were bactericidal to the clinical strain at 500 μg/mL (Table 2).

**Table 1:** Antibacterial activity towards MRSA of *B. kockiana* flower sequential extracts (1.0 mg/disc) evaluated using disc diffusion assay

Extracts	Diameter of inhibition zone (mm)	
	MRSA ATCC 33591	MRSA Clinical isolate
Hexane	–	–
Dichloromethane	–	–
Ethyl acetate	11.7 ± 0.7	11.3 ± 0.7
Methanol	11.0 ± 0.0	11.3 ± 0.7
Vancomycin^a^	21.0 ± 0.0	21.0 ± 0.0

Note: diameter of the bacterial disc is 6 mm. Results are expressed as means ± SD obtained from 3 independent experiments. ^a^Vancomycin used in 0.03 mg/disc. “-” represents no activity

**Table 2:** Minimum inhibitory concentration (MIC), minimum bactericidal concentration (MBC) and phytochemical content of *B. kockiana* flower ethyl acetate and methanol extracts

Extracts	Phytochemical content	MIC (μg/mL)		MBC (μg/mL)	
		MRSA ATCC 33591	MRSA Clinical isolate	MRSA ATCC 33591	MRSA Clinical isolate
Ethyl acetate	Tannins	125	62.5	125	500
Methanol	Tannins, flavonoids	250	125	250	500
Vancomycin	n.t	1.00	2.00	n.t	n.t

Note: “n.t” represents not tested

### Structural elucidation of compounds 1 and 2

The chemical structure of the components isolated from *B. kockiana* flower was accomplished by comparing mass spectrum, UV spectrum, IR, ^1^H–NMR and ^13^C–NMR data with literature. Compound **1** isolated from Fraction FE2–3 was identified as gallic acid (IUPAC name is 3,4,5-trihydroxybenzoic acid; molecular formula C_7_H_6_O_5_). It was isolated as yellow needles which melted at 250–252 °C. It appeared as one spot with R_*f*_ value 0.77 on thin layer chromatography (TLC) in acetone:ethyl acetate (6:4) solvent system, and the spot turned ferric chloride solution to dark blue, indicating the presence of phenol group. Mass spectrometry showed that it has a molecular ion peak of m/z 170 (100%), and other major fragments are seen at 153 (95%), and 125 (20%) (Additional file 1: Figure S1). This was in agreement with the fragmentation pattern of an aromatic carboxylic acid. The fragmentation pathway of an aromatic carboxylic acid is the loss of OH to form C_6_H_5_O_3_CO^+^ (m/z = 153), followed by loss of CO to form C_6_H_5_O_3_ (m/z = 125). The absorption spectrum of gallic acid exhibits two peaks in the UV range, at 230 and 270 nm (Additional file 2: Figure S2). The structure is further substantiated by the IR spectrum, where a broad peak at 3000–3400 cm^− 1^ (O-H, H-bonded), sharp peaks at 1690 cm^− 1^ (C=O stretch), 1600–1680 cm^− 1^ (aromatic C=C stretch) and 1260 cm^− 1^ (C-O stretch) are observed (Additional file 3:Figure S3). ^1^H–NMR spectrum of gallic acid showed only one signal at δ_H_ 7.15 (2H, *s*, H-2, H-6) (Additional file 4: Figure S4) while ^13^C–NMR spectrum showed five signals: δ_C_ 145.9 (C-3, C-5), δ_C_ 138.6 (C-4), δ_C_ 121.9 (C-1), δ_C_ 110.0 (C-2, C-6), and δ_C_ 167.8 for carbonyl group (Additional file 5: Figure S5). The spectra data were in agreement with that for gallic acid reported [21].

Compound **2** isolated from FE2–3-4 was identified as methyl gallate (IUPAC name is methyl 3,4,5-trihydroxybenzoate; molecular formula C_8_H_8_O_5_). It was isolated as white crystal, with melting point 199–201 °C. It appeared as one spot on the TLC plate with R_*f*_ value 0.77 developed using acetone:methanol:chloroform (4:2:4) solvent system. The spot turned to dark blue when sprayed with ferric chloride reagent, showing the presence of phenol group. Mass spectrometry showed that methyl gallate has a molecular ion peak of m/z 184 (80%), and other major fragments at 153 (100%) and 125 (95%) (Additional file 6: Figure S6). It is compatible to the fragmentation pathway of an alkyl benzoate ester, where the the alkoxy group is first lost to form C_6_H_5_O_3_CO^+^ (m/z = 153), followed by loss of CO to form C_6_H_5_O_3_ (m/z = 125). Two maximum absorption peaks are present in the UV spectrum, at 218 and 274 nm (Additional file 7: Figure S7). The chemical structure is also supported by the IR spectrum, with the absorption bands appeared at 3480 cm^− 1^ (OH stretch, intermolecular H-bond), 1700 cm^− 1^ (C=O stretch) and 1600–1680 cm^− 1^ (aromatic C=C stretch) (Additional file 8:Figure S8). The ^1^H–NMR spectrum showed two major signals: a methoxyl signal at δ_H_ 3.77, and aromatic protons signal at δ_H_ 7.10 (2H, *s*, H-2, H-6) (Additional file 9: Figure S9) while the ^13^C–NMR spectrum showed six signals: δ_C_ 146.0 (C-3, C-5), δ_C_ 138.7 (C-4), δ_C_ 121.6 (C-1), δ_C_ 109.7 (C-2, C- 6), δ_C_ 167.1 for carbonyl group and δ_C_ 51.8 for methoxy group (Additional file 10: Figure S10). The spectra data obtained are in agreement with that of methyl gallate reported [22, 23].

### Antibacterial activity of compound 1 and 2 towards MRSA

The antibacterial potency of compound **1** and **2** were examined and results showed that both could inhibit the growth of MRSA (Table 3). Ethyl acetate extract exhibited stronger inhibition activity than compound **1** and **2** in both ATCC and clinical strains. Since compound **1** and **2** were less potent than extract and positive control, therefore both compounds required higher concentration to inhibit the growth of MRSA. Clinical isolate was more susceptible (lower MIC) to extracts t (Table 2), but MRSA ATCC 33591 is more susceptible to compound **1** and **2** (Table 3).

**Table 3:** Minimum inhibitory concentration (MIC) of gallic acid and methyl gallate against MRSA

Compounds	MIC (μg/mL)	
	MRSA ATCC 33591	MRSA (Clinical isolate)
**(1)**	300	600
**(2)**	250	500
Ethyl acetate extract	125	62.5
Vancomycin	1.00	2.00

**Fig. 4:**
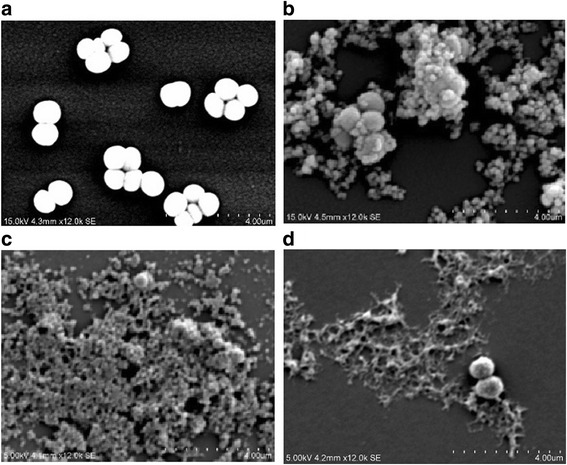
Scanning electron microscopy (SEM) photograph of MRSA treated with ethyl acetate and isolated active compounds of *B. kockiana* flower at 2 × MIC (**a**) Control (**b**) treated with ethyl acetate extract (**c**) treated with compound **1** (**d**) treated with compound **2.**

In SEM analysis, the micrographs revealed that untreated MRSA appeared as cocci, grape-like cluster or chain, smooth surface and plump appearance. The membrane layer of the bacterial cells were disrupted upon treatment with 2 × MICs of ethyl acetate extract and compounds (Fig. 4). Bacterial cell membrane was severely disrupted with evident plasmolysis upon treatment. Massive leakage of cell content was observed in all treated cells.

## Discussion

Antibacterial activity of *B. kockiana* flowers and leaves have been previously reported in Chew et al. [15]. Our preliminary study reported that the flower extract exhibited fairly strong antibacterial activity towards various Gram positive bacteria, namely *Bacillus cereus*, *Micrococcus lutues*, methicillin sensitive *S. aureus* (MSSA) and MRSA. Sequential extraction was performed in flower samples, using organic solvents of increasing polarities to extract various classes of phytochemicals according to their polarities. Extraction using hexane removed the waxes, fats, lipids and fixes oils; dichloromethane extracted most of the chlorophyll, aglycones and volatile oils; ethyl acetate and methanol extracted the polar chemical constituents, i.e. flavonoids, tannins, glycosides, and polyphenols.

This is the first report on the antibacterial compounds isolated from *B. kockiana*. Tannins and flavonoids detected in phytochemical screening correlated well with the class of compounds isolated. Lower MIC and MBC in ethyl acetate extract could be explained due to the presence of other tannins and phenols (not isolated) which could possibly contribute to inhibitory activity of the extract. It is interesting to observed that the susceptibility of MRSA strains was different in extracts and compounds. The potency of compound 1 and 2 decreased when they are tested individually as pure compounds. This could be due to the presence of numerous constituents that could potentially causing synergistic multi-target effects toward the antibacterial activity [24]. Synergistic multi-target effects are the multitargeted strategy in combining herbals and drugs to act on several targets. Phytotherapy which based on the combined action of a mixture of constituents. The mixture of constituents may act on several antibacterial targets concurrently, i.e. depolarizing the cell membrane, inhibiting the efflux pump, disintegrating the genetic materials [24–26]. T Efferth and E Koch [25] commented that the presence of multiple constituents could exhibit better activity on host, compare to a single compound. Loss of effect during bioactivity-guided isolation is possible because the synergistic multitarget antibacterial effects were vanished when the single compound was isolated. This could best explain our findings on the antibacterial potency of extracts and compounds isolated in this study. Numerous studies have reported that plant polyphenols could synergistically interact with each other to exhibit stronger antibacterial activities [25, 35, 36]. Potency of phytochemicals could be improved when they are combined in treatment, since multiple sites in bacteria are targeted. Phytochemicals could alter the outer membrane permeability, inhibit multidrug resistance efflux pumps, function as active sites modifiers and β-lactamase inhibitors [36, 37]. Basri et al. found that antibacterial activity of oxacillin against MRSA is stabilised with gallic acid, as gallic acid will function as additive [35]. Combination of oxacillin with alkyl gallates (methyl to decyl gallates) could result in stronger inhibitory effect [25]. Hemaiswarya and Doble discovered that synergistic effect of phenolic acids, i.e. cinnamic, chlorogenic, and caffeic acids were associated with membrane damaging activity, where they would target on the hydrophobic bacterial surface, modify the membrane fluidity and disrupt the cell membrane structure [27]. Ampicillin and vancomycin could inhibit bacterial cell wall synthesis. It was reported that the potency of ampicillin and vancomycin can be enhanced if they were administered together with the phenolic acids, in a non-specific manner [27].

Literatures reported that the potency of compound 1 and its ester derivatives was structurally related. The antibacterial potency against MRSA increases with the alkyl chain length of the ester derivatives, where lower MIC and MBC were noticed [28, 29]. Kubo et al. reported that gallic acid inhibits the growth of MRSA ATCC 33591 at MIC value 3200 μg/mL and MBC value > 3200 μg/mL, which is higher than the MIC value obtained in this study [28]. Reduction in MIC and MBC with increasing alkyl chain length has been reported [28, 29]. MRSA inhibition activity was maximised when the alkyl chain length was C_10_ (decyl gallate) [29]. Shibata et al. showed that decyl (C_10_), undecyl (C_11_) and dodecyl (C_12_) gallates exhibited the strongest inhibition activity against MRSA (MIC 12.5 μg/mL and MBC 25 μg/mL) [29]. This is evident that alkyl chain is extremely important in exhibiting the MRSA inhibition activity. The longer the alkyl chain, the stronger the inhibition towards MRSA. Various studies have reported that alkyl chain length was extremely crucial in exhibiting antibacterial and antifungal activities [30–32]. The optimal chain length is dependent to the target species and the mechanism of action. For instance, anti-salmomella activity is the strongest in nonyl (C_9_) gallate. Similar alkyl chain length (C_9_ and C_10_) was also reported to exhibit the strongest antibacterial activity against *Bacillus subtilis* [32]. However, antifungal activity of alkyl gallate was the strongest in octyl gallate (C_8_) [30]. Octyl gallates exhibited the strongest antifungal activity in numerous fungus, namely *Chaetomium globosum*, *Wolfiporia extensa*, *Gleophyllum traveum*, *Trametes versicolor*, *Lenzities betulina*, *Saccharomyces cerevisiae*, *Zygosaccharomyces bailii*, *Candida albicans*, and *Aspergillus niger* [30, 31].

Bacterial membrane was severely disrupted upon the treatment, where significant cell plasmolysis was noticed. This showed that antibacterial compounds in ethyl acetate extracts, including compound 1 and 2 are mostly acted on the bacterial cell membrane, causing extensive damages to MRSA. This finding provides useful clue to phytochemicals to be discovered from this plant in near future. Compound 1 and 2 have similar structures and functional groups. Both consist of the pyrogallol moiety: three hydroxyl groups covalently linked to a benzene ring, where part of the molecule gains the hydrophilic character. The presence of pyrogallol moiety and alkyl chain/carboxylic group resulted in amphiphilic nature of the molecules. Amphiphilic nature of phytochemicals is closely associated to the molecular interaction with the cell, attachment to cell membrane and penetration into the cell. To elicit the antibacterial action towards MRSA, the hydroxyl groups of pyrogallol moiety of compound 1 and 2 would form intermolecular hydrogen bonds with phospholipid polar head of the bacterial membrane, and also in deeper regions by hydrophobic interaction with the acyl chains [33, 34]. The hydrophobic alkyl chain would then bend towards the membrane, disrupt the membrane fluidity, and alter the membrane permeability [29, 35, 36]. Amphiphilic drugs that possess polar and non-polar moieties could establish molecular interaction with lipid acyl chain at the outer membrane phospholipid and some can act on membrane bound proteins [29, 37]. Similar cell morphology viewed under SEM was also reported when *K. pneumoniae* was treated with total tannins and ethyl gallate [38]. Recent study reported by Król et al. stated that alkyl gallates could interfere in cell division [36]. Alkyl gallates bind tightly to cytoplasmic protein *FtsZ* and inhibit the polymerisation [36]. Cell division was terminated when the assembly of Z-ring at the site of division is inhibited.

Besides, alkyl gallates could interfere the bacterial respiratory systems, i.e. inhibition of oxygen consumption and NADH oxidase, specifically target on the electron transport chain, and redox reactions [31, 32]. Alkyl gallates could act as a pro-oxidant, induce the generation of reactive oxygen species (ROS). ROS generated could oxidise the unsaturated fatty acids in the membrane lipid layer, resulting in reduction in membrane fluidity, disruption of membrane structure and functions [31].

## Conclusion

Our findings showed that gallic acid and methyl gallate were present in *B. kockiana* flower and they both could exhibit antibacterial activity towards MRSA. The study of antibacterial mechanisms using SEM revealed the mode of action of extract and compounds based on the morphological observation of the cells.

## Additional files

Additional file 1: **Figure S1.** GA MS – Mass spectrum of gallic acid – Gas chromatography mass spectrum of gallic acid. (JPEG 27 kb)

Additional file 2: **Figure S2.** GA UV – UV-Vis of gallic acid – Ultraviolet-visible spectrum of gallic acid. (JPEG 23 kb)

Additional file 3: **Figure S3.** GA IR – FTIR of gallic acid – Fourier transform infrared spectrum of gallic acid. (JPEG 23 kb)

Additional file 4: **Figure S4.**GA HNMR – 1H NMR of gallic acid – ^1^H nuclear magnetic resonance spectrum of gallic acid. (JPEG 21 kb)

Additional file5: **Figure S5.** GA CNMR – 13C NMR of gallic acid – ^13^C nuclear magnetic resonance spectrum of gallic acid. (JPEG 24 kb)

Additional file 6: **Figure S6.** MG MS – Mass spectrum of methyl gallate – Gas chromatography mass spectrum of methyl gallate. (JPEG 26 kb)

Additional file 7: **Figure S7.** MG UV – UV-Vis of methyl gallate – Ultraviolet-visible spectrum of methyl gallate. (JPEG 27 kb)

Additional file 8: **Figure S8.** MG IR – FTIR of methyl gallate – Fourier transform infrared spectrum of methyl gallate. (JPEG 24 kb)

Additional file 9: **Figure S9.** MG HNMR – 1H NMR of methyl gallate – ^1^H nuclear magnetic resonance spectrum of methyl gallate. (JPEG 22 kb)

Additional file 10: **Figure S10.** MG CNMR – 13C NMR of methyl gallate – ^13^C nuclear magnetic resonance spectrum of methyl gallate. (JPEG 26 kb)

## Abbreviations

cfu: coliform forming unit
FTIR: fourier transform infra-red
MBC: minimum bactericidal concentration
MIC: minimum inhibitory concentration
MRSA: methicillin resistant *Staphylococcus aureus*
MSSA: methicillin sensitive *Staphylococcus aureus*
NMR: nuclear magnetic resonance;
PBS: phosphate buffer saline
ROS: reactive oxygen species.
SEM: scanning electron microscopy
TLC: thin layer chromatography
VRSA: vancomycin resistant *Staphylococcus aureus*
