# Supplementary figures and images for: Anti-methicillin-resistance Staphylococcus aureus (MRSA) compounds from Bauhinia kockiana Korth. And their mechanism of antibacterial activity
Source: BMC Complement Altern Med. 2018 Feb 20;18:70. doi: 10.1186/s12906-018-2137-5 (PMC5819667; doi:10.1186/s12906-018-2137-5)

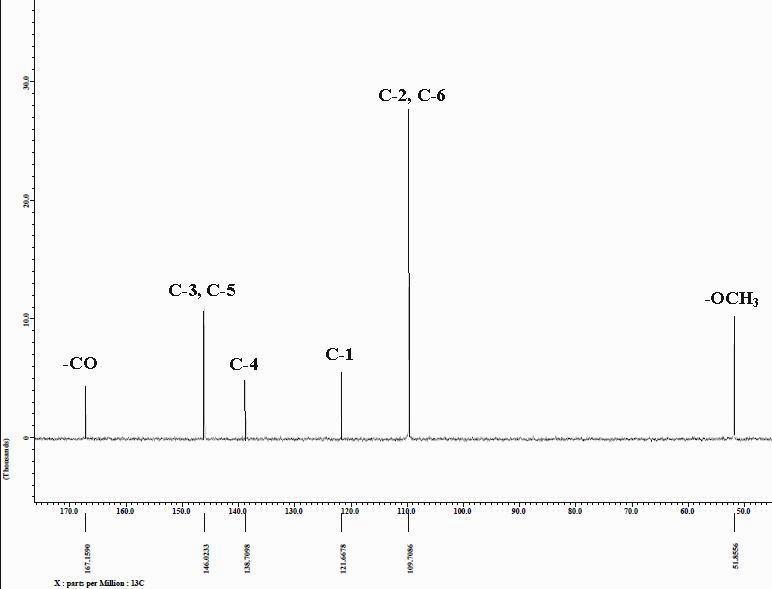

Supplement: Supplementary file 1 — Figure S1. GA MS – Mass spectrum of gallic acid – Gas chromatography mass spectrum of gallic acid. (JPEG 27 kb) [file 12906_2018_2137_MOESM10_ESM.jpg]

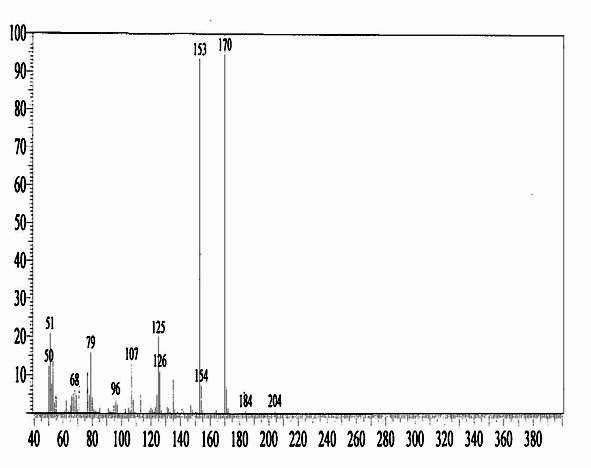

Supplement: Supplementary file 2 — Figure S2. GA UV – UV-Vis of gallic acid – Ultraviolet-visible spectrum of gallic acid. (JPEG 23 kb) [file 12906_2018_2137_MOESM1_ESM.jpg]

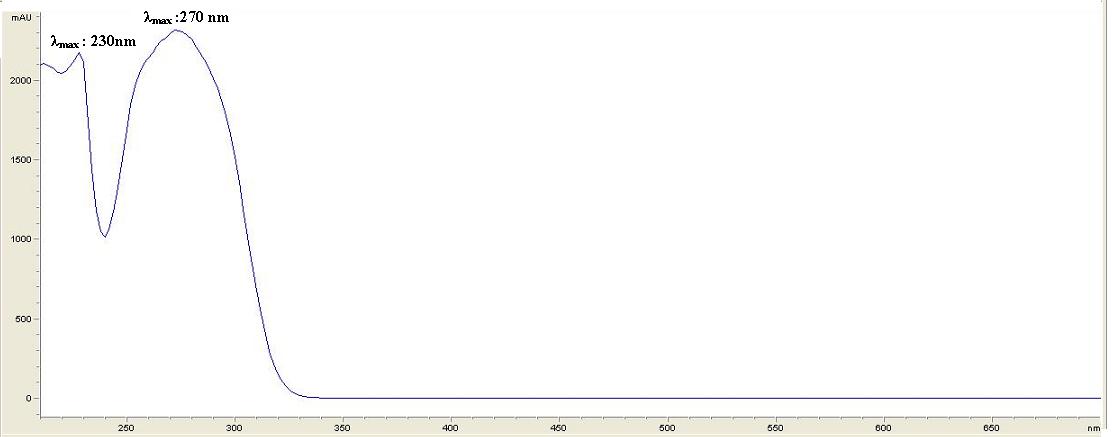

Supplement: Supplementary file 3 — Figure S3. GA IR – FTIR of gallic acid – Fourier transform infrared spectrum of gallic acid. (JPEG 23 kb) [file 12906_2018_2137_MOESM2_ESM.jpg]

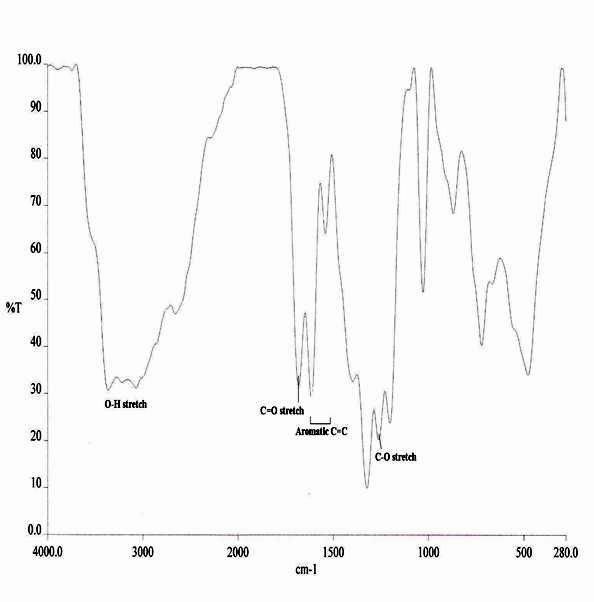

Supplement: Supplementary file 4 — Figure S4.GA HNMR – 1H NMR of gallic acid – 1H nuclear magnetic resonance spectrum of gallic acid. (JPEG 21 kb) [file 12906_2018_2137_MOESM3_ESM.jpg]

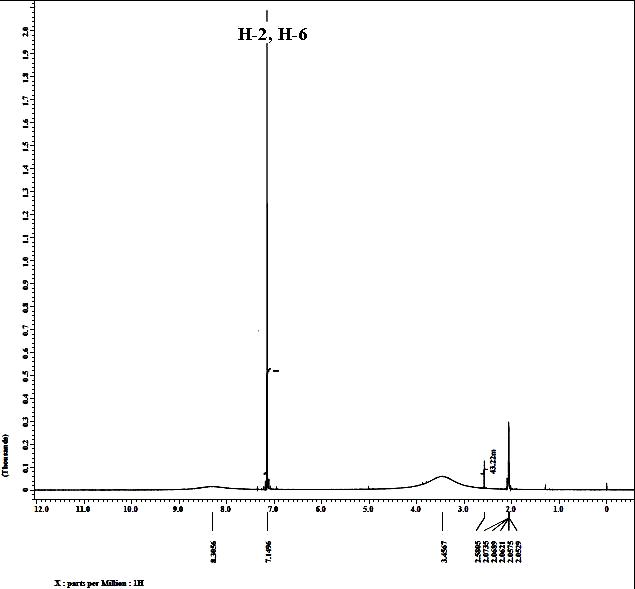

Supplement: Supplementary file 5 — Figure S5. GA CNMR – 13C NMR of gallic acid – 13C nuclear magnetic resonance spectrum of gallic acid. (JPEG 24 kb) [file 12906_2018_2137_MOESM4_ESM.jpg]

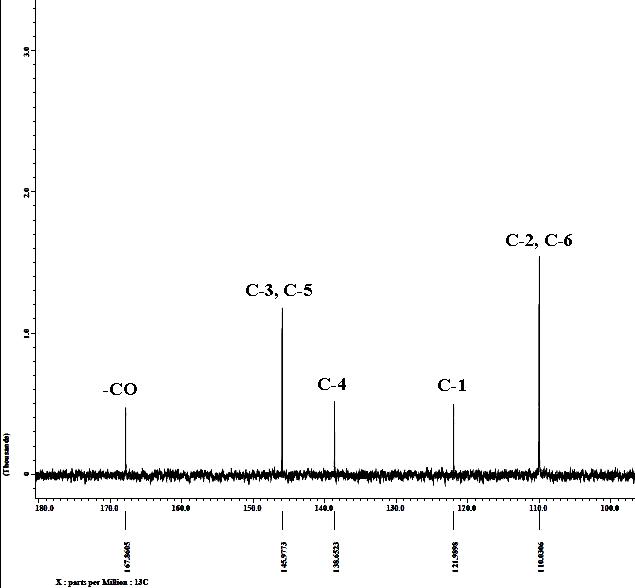

Supplement: Supplementary file 6 — Figure S6. MG MS – Mass spectrum of methyl gallate – Gas chromatography mass spectrum of methyl gallate. (JPEG 26 kb) [file 12906_2018_2137_MOESM5_ESM.jpg]

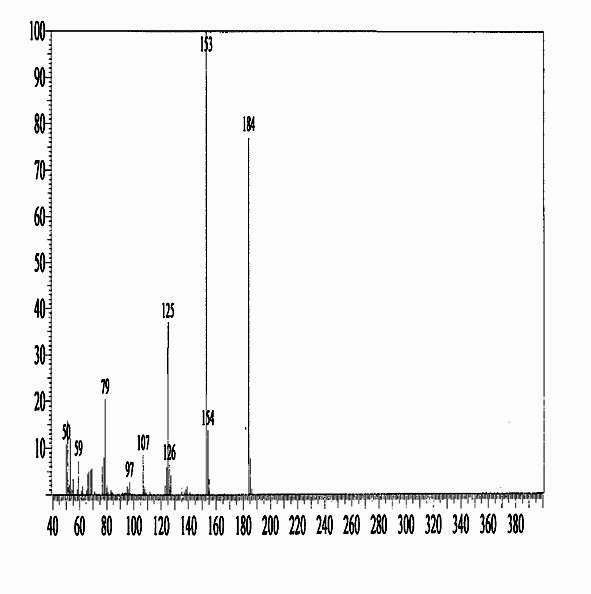

Supplement: Supplementary file 7 — Figure S7. MG UV – UV-Vis of methyl gallate – Ultraviolet-visible spectrum of methyl gallate. (JPEG 27 kb) [file 12906_2018_2137_MOESM6_ESM.jpg]

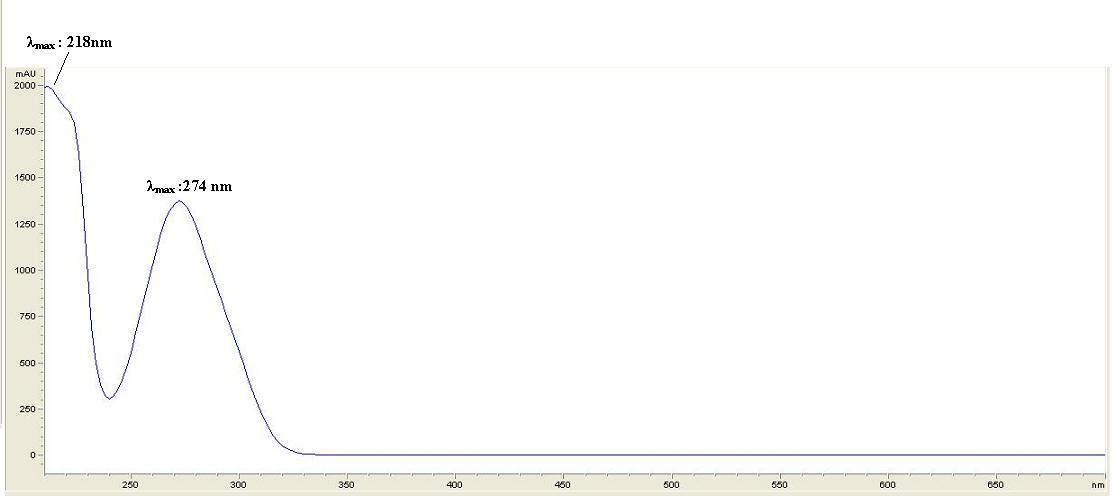

Supplement: Supplementary file 8 — Figure S8. MG IR – FTIR of methyl gallate – Fourier transform infrared spectrum of methyl gallate. (JPEG 24 kb) [file 12906_2018_2137_MOESM7_ESM.jpg]

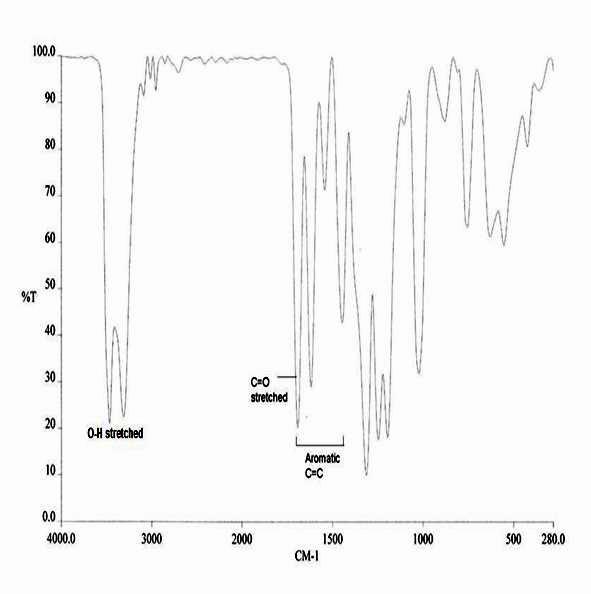

Supplement: Supplementary file 9 — Figure S9. MG HNMR – 1H NMR of methyl gallate – 1H nuclear magnetic resonance spectrum of methyl gallate. (JPEG 22 kb) [file 12906_2018_2137_MOESM8_ESM.jpg]
